# The IDO inhibitor 1-methyl tryptophan activates the aryl hydrocarbon receptor response in mesenchymal stromal cells

**DOI:** 10.18632/oncotarget.20166

**Published:** 2017-08-10

**Authors:** Holly C. Lewis, Raghavan Chinnadurai, Steven E. Bosinger, Jacques Galipeau

**Affiliations:** ^1^ Departments of Pediatrics and Hematology & Medical Oncology, Emory University School of Medicine, Atlanta, GA, USA; ^2^ Department of Medicine and University of Wisconsin Carbone Cancer Center, University of Wisconsin in Madison, Madison, WI, USA; ^3^ Department of Pathology & Laboratory Medicine, Emory University, Atlanta, GA, USA; ^4^ Yerkes NHP Genomics Core Laboratory, Div. Microbiology & Immunology, Yerkes National Primate Research Center, Atlanta, GA, USA

**Keywords:** MSC, AHR, IDO, 1-methyl tryptophan, cancer immunotherapy

## Abstract

The catabolism of tryptophan (Trp) by indoleamine 2,3-dioxygenase (IDO) is a key step in tolerance effected by a variety of cell types, including mesenchymal stromal cells (MSCs). Trp catabolism generates molecules known as kynurenines, whose tolerance mechanisms involve activation of the Aryl Hydrocarbon Receptor (AHR). A synthetic analog of Trp, 1-methyl tryptophan (1MT), is a selective inhibitor of IDO enzymatic activity being utilized in cancer immunotherapy trials. We hypothesized 1MT might activate AHR independently of its effects on IDO. We demonstrate MSCs express AHR protein, and that *in vitro* treatment with 1MT causes AHR nucleotranslocation. Upon analyzing mRNA, we observed transcriptional upregulation of cytochrome p450 1a1 and 1b1 by 1MT racemic mixture (R-MT), consistent with AHR-activation. RNA-sequencing identified Nrf2, MAPK12 and IL-1a as downstream targets of 1MT. We demonstrate 1a1 and 1b1 activation by 1MT in IDO+ MSC following interferon-γ (IFN-γ) activation, suggesting AHR signaling is uncoupled from IDO catalytic function. Such a mechanism of action for 1MT may extend its usage to a wider range of patients, irrespective of tumor IDO expression. These observations support a novel paradigm by which AHR-activating compounds like 1MT can be used in cancer immunotherapy to stimulate a pro-inflammatory response.

## INTRODUCTION

Recent studies in cancer immunology have explored the role of tolerance inside the tumor microenvironment, enabling cancers to evade immune surveillance [1]. Cells that mediate tumor-associated suppression include myeloid suppressor cells or tumor-associated macrophages. Such cells have been shown to facilitate tumor progression by the accumulation of regulatory T cells [1]. One of the principle mechanisms whereby tumor-resident cells mediate this immunomodulation is the catabolism of tryptophan (Trp) by indoleamine 2,3-dioxygenase (IDO). It has been shown that IDO is a crucial determinant of the immunomodulatory abilities of mesenchymal stromal cells (MSCs) [2]. Immune-suppressing cells with IDO expression engender a tolerogenic tumor microenvironment [3] providing a rationale for pharmacologically blocking IDO activity with 1MT for cancer immunotherapy. IDO catalytic activity leads to the deprivation of Trp and has been shown in biochemical studies to dampen the proliferation of T cells by limiting ζ-chain activation [4]. However, the Trp-deprivation model has been questioned by studies showing IDO-catalyzed Trp catabolites bind to and activate the aryl hydrocarbon receptor (AHR) [5]. Much of our understanding of aryl hydrocarbons comes from studies with 2,3,7,8-tetrachlorodibenzodioxin (TCDD). First described as the TCDD receptor, ligand-activation of AHR causes a conformational shift, allowing it to bind its chaperone protein, AHR nuclear translocator (ARNT). ARNT contains a nuclear-localization-signal (NLS) in residues 39-61 [6] which allows the complex entry to the nucleus, whereupon it activates transcription at AHR response elements (AHREs) [1, 7]. Signaling at AHREs has been implicated in carcinogenesis studies with aromatic hydrocarbons like benzopyrene [8, 9]. In such studies, ligand-activation of AHR is often shown by the upregulation of cytochrome p450 (Cyp) enzymes, Cyp1a1 and Cyp1b1 [10]. However, the evolutionary conservation of AHR signaling (including invertebrates with no such hepatic biotransformation of toxins [11]) suggests a broader homeostatic function for AHR signaling, beyond just toxin-processing. Indeed, the finding that endogenous kynurenines can activate the AHR suggests this transcription factor may have broadly-acting immunomodulatory effects [12]. Like Trp and Kyn, 1MT is also an aromatic hydrocarbon, but 1MT is currently the focus of more than a dozen clinical oncology trials [13], where its use is rationalized on the basis of its irreversible inhibition of IDO catalysis. Since immune-competent cells, such as MSCs and dendritic cells, can co-express AHR and IDO under inflammatory conditions [14], it suggests that the effects of 1MT ascribed to selective inhibition of IDO may also arise from activation of the AHR pathway. We here demonstrate that AHR+ MSCs with IDO competency deploy a robust inflammatory molecular genetic response to 1MT, even in the absence of IDO expression. These data provide important insights that may expand the clinical indications for 1MT as a cancer immunotherapy, suggesting that it may be therapeutic even in IDO-null tumors, through activating AHR-mediated mechanisms.

## RESULTS

### Immunophenotype of marrow-derived human mesenchymal stromal cells

**Figure 1:**
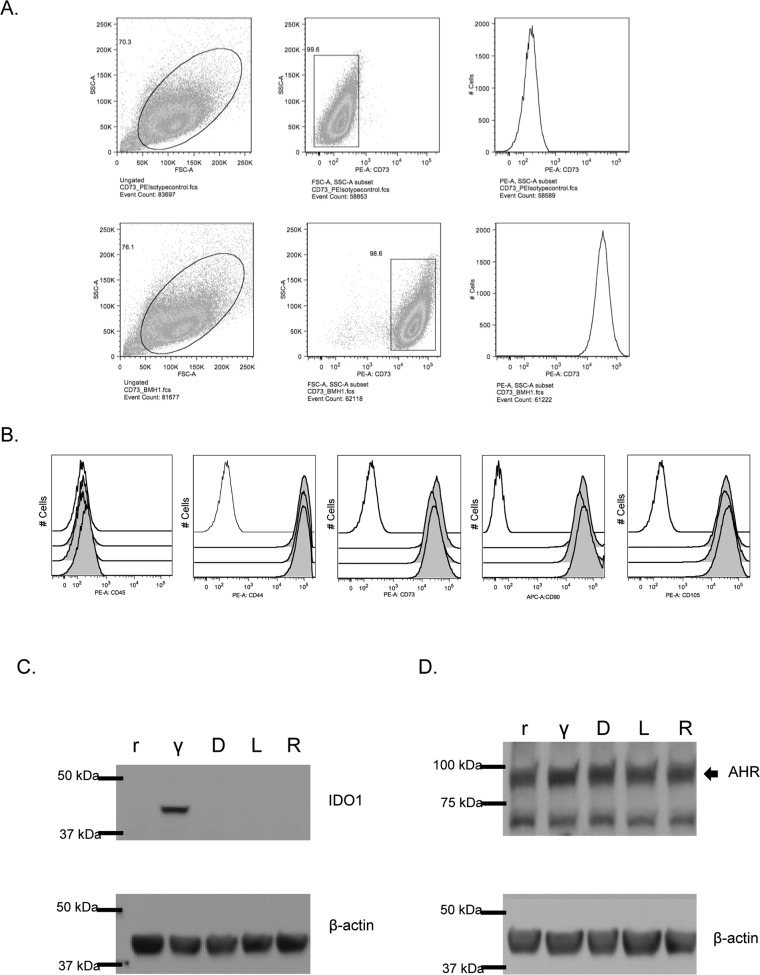
IDO and AHR expression in resting and IFN-γ-stimulated MSC treated with 1MT **(A)** Mesenchymal stromal cells (MSC) were isolated from the marrow of healthy human donors (N=3). Cells were removed from flasks after 5d of growth and stained using a panel approved by the International Society for Cell Therapy. Panel A represents the flow cytometry gating strategies for an isotype control sample, compared to an MSC sample stained with PE-conjugated CD73. These data are representative of all MSC samples utilized in this study. **(B)** Panel B represents the sub-gating analysis, interrogating MSC for CD45, CD44, CD73 and CD90 and CD105. In each histogram, the black unfilled-line represents relevant isotype-matched control, and the three gray lines are independent but contemporaneously-analyzed MSC samples. **(C)** Untreated, resting (r) MSCs and IFN-γ stimulated (γ), MSCs were analyzed for expression of the IDO protein (IFN-γ: 50 ng/ml for 24 h). Additionally, treatment with (D)-1MT, (L)-1MT or racemic (R) mixture was tested (1 mM each). Figure 1C represents the immunoblotting results of a single membrane that was first blotted for IDO1, then stripped, re-blocked and probed for actin. These are results from an experiment with MSC sample, which was replicated three times. (D) At baseline, resting MSCs (r) demonstrate presence of the AHR protein. The effects of 24h treatment with IFN-γ (γ), or D-MT, L-MT or R-MT on AHR protein expression was evaluated. IFN-γ: 50 ng/ml; all 1MT: 1mM). AHR is indicated by the arrowhead near the 100 kDa band. Figure 1D epresents the immunoblotting results of a single membrane that was cut into two and blotted separately for AHR and actin. These are results from an experiment with one MSC sample, which was replicated three times.

We performed flow cytometry to confirm that MSCs expressed conventional cell surface markers, using guidelines from the International Society for Cellular Therapy [15]. Figure 1A presents the flow cytometry gating strategy used to confirm the presence of these markers for one MSC donor, in comparison with relevant matched-isotype control samples. Figure 1B compares three distinct MSC samples, analyzed using the same gating strategy. These findings are representative of all MSCs used in subsequent analyses.

### MSCs constitutively express AHR but inducibly express IDO

The Trp derivative 1MT has been classically described as an enzymatic inhibitor of the IDO1 enzyme. As IDO is an important protein for MSC function, we sought to assess the effects of 1MT on MSCs. Resting MSCs (rMSCs) are immunoregulatory at baseline, but not nearly as effective as MSCs that have been pre-licensed with inflammatory stimuli such as interferon-γ (IFN-γ). IFN-γ activates a STAT1-mediated signaling cascade that causes de novo mRNA transcription and protein expression of IDO1 [16]. Figure 1C confirms this, showing that rMSCs are IDO-negative and that IFN-γ induces robust IDO protein upregulation. Treatment with any of the enantiomer mixtures of 1MT does not induce IDO expression (Figure 1C). As the AHR protein has been described as being constitutively present in the cytoplasm at baseline [17], we sought to confirm that our MSCs expressed this protein. Figure 1D summarizes these findings, in which the antibody localizes the AHR protein near the 100 kDa marker. Notably, these two immunblots demonstrate that 1MT alone does not induce IDO expression, nor alter the level of AHR expression.

### 1MT causes AHR nucleotranslocation

**Figure 2:**
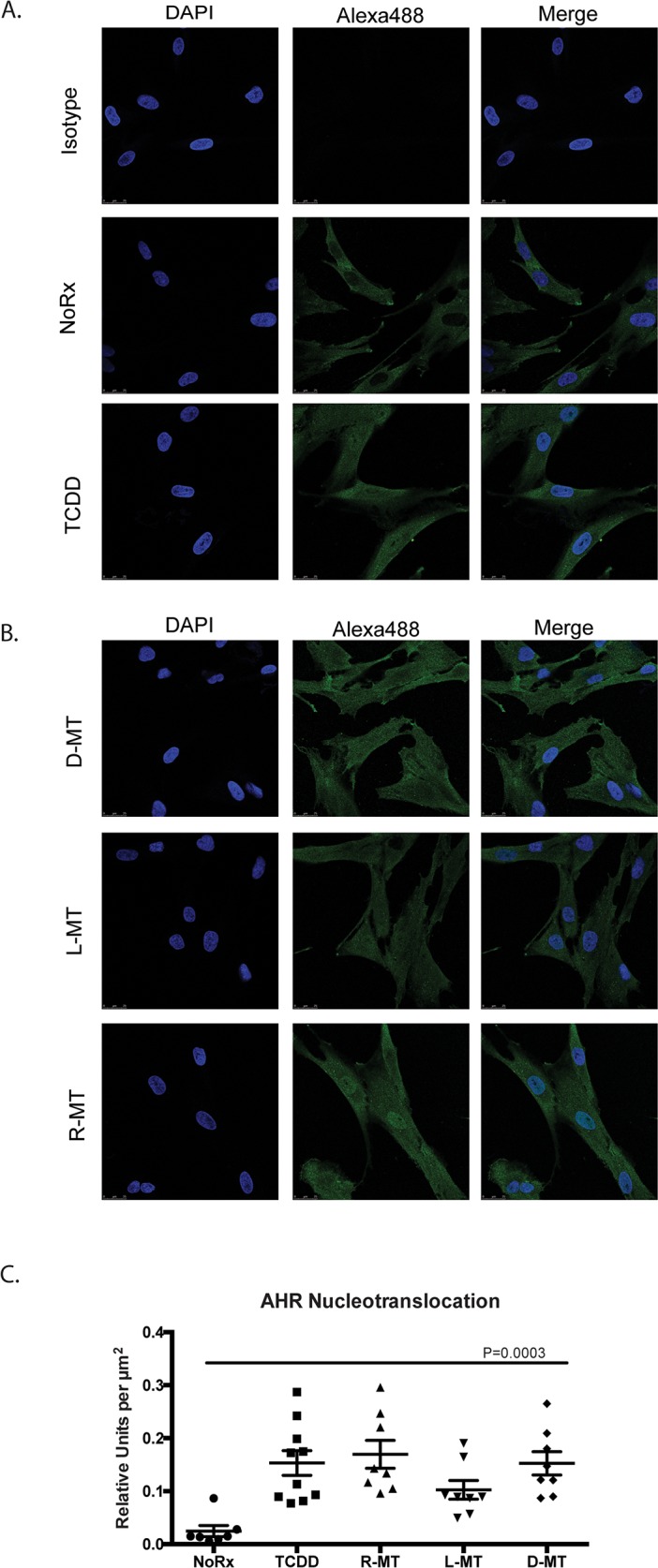
AHR nucleotransloaction in MSCs treated with 1MT and AHR agonists **(A, B)** MSCs were plated onto glass coverslips and allowed to adhere overnight. Media was aspirated and replaced with R10 or the indicated drug. Drugs were left on cells for 5h, after which cells were fixed and AHR was visualized via immunofluorescence; DAPI was used to visualize nuclei. Isotype-control was a non-specific murine-derived IgG. TCDD concentration was 10 nM. Concentrations of D-MT, L-MT, R-MT all at 1mM. These results **(A, B)** from an experiment with one MSC sample, which was replicated four times with independent MSC samples. All images were taken using a confocal microscope with the same exposure settings. **(C)** The bar graph represents the quantified results of nucleotranslocation, as observed via immunofluorsence. The Leica LASX software package was utilized to delimit regions of interest, defined by the DAPI-visualized nucleus. From these regions, the signal of Alexa488 was computed and normalized per μm^2^. These data are the cumulative average of three experiments using independent MSC samples, each with an average of twelve enumerations per high-power field. Statistical test performed was one-way ANOVA, P=0.003.

Upon ligand binding, the AHR associates with ARNT, only upon which will the protein enter the nucleus, where it acts as a transcription factor at AHREs. To generate evidence that 1MT could induce this pathway of activation, we used a protein-based tracking method, to document a shift of AHR protein from cytoplasm-to-nucleus, after treatment with test drugs [18, 19]. Figures 2A-2B demonstrate that at baseline, MSCs exhibit a cytoplasmic signal for AHR, and nuclei that are devoid of the green immunofluorescent signal. This is readily observed when comparing the untreated cells (NoRx) to the isotype-stained cells (Isotype). After 5h of TCDD treatment, an increase in nuclear-staining can be appreciated, consistent with its classification as a bona fide AHR ligand. We performed this experiment using three enantiomeric mixtures of 1MT, and then utilized Leica software packages to numerically quantify the resultant changes in immunofluorescence (Figure 2C). We performed a one-way ANOVA test, affording a p-value of 0.0003, indicating that the nuclear shift in AHR signal induced by 1MT was comparable to that induced by TCDD. Taken together, these data demonstrate 1MT activates a similar cellular response as the most well-understood AHR ligand.

### Known AHR ligands and trp derivatives activate the AHR response

As discussed above, bona fide AHR ligands bind the molecule and activate its nucleotranslocation, resulting in the induction of genes that contain an AHRE. The most well-characterized sentinel genes of such AHR activation are Cyp1a1 and Cyp1b1 [8, 20, 21]. We cultured MSCs in the presence of two validated AHR ligands, TCDD and 6-formylindolo [3,2-b]carbazole (FICZ), well-characterized molecules known to ligate the receptor [22]. Additionally, we included two IDO-catabolized Trp byproducts, kynurenine and kynurenic acid, both of which have been explored for their AHR bioactivity. Kynurenic acid in particular has been documented as a verified AHR ligand that results in more potent cytochrome induction that kynurenine [23]. Untreated controls were included in each experiment, and Fold-Induction of each Cyp gene was calculated relative to baseline expression of GAPDH. Figure panels 3A-3D plot the induction of Cyp1a1 and Cyp1b1 following 6h or 24h timepoints. We note that racemic 1MT (R-MT) induces significant induction for Cyp1a1, and that the other test ligands responded with the prototypic AHR response. Although the induction of Cyp1b1 by R-MT did not achieve statistical significance, we note that the magnitude of cytochrome induction for known ligands FICZ and kynurenic acid are similar to that effected by racemic 1MT.

### 1MT induces dose-dependent response for AHR activation in MSCs

**Figure 3:**
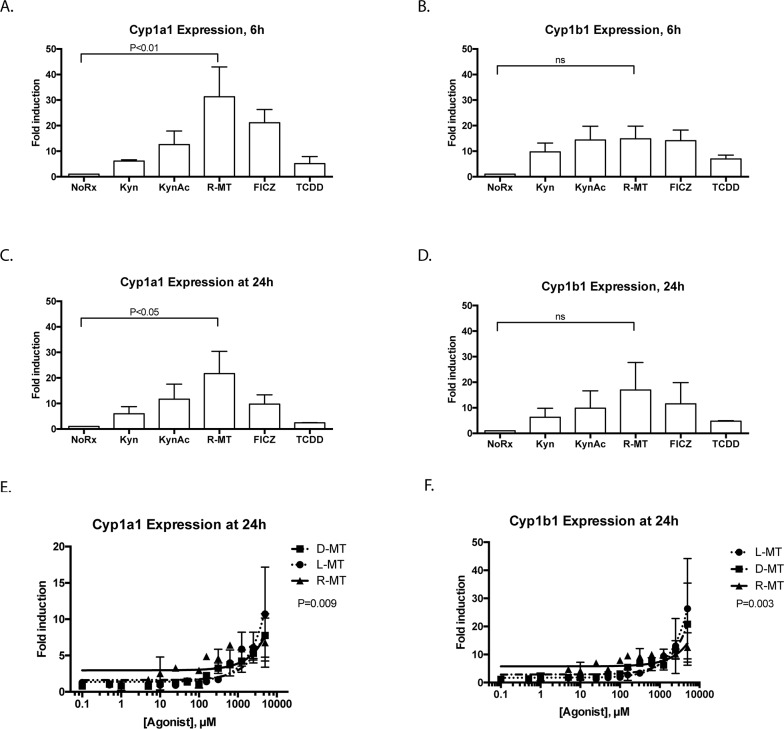
Known AHR ligands and Trp derivatives activate the AHR response in MSCs **(A-D)** MSCs were cultured in the presence of well-characterized AHR-binding ligands or other derivatives of tryptophan. Concentrations used in these fixed-dose studies were FICZ, TCDD: 10nm; Kyn, KynAc: 500 μM, 1MT: 1mM. After 6 or 24h, cells were harvested, lysed and total RNA was extracted, converted to cDNA and analyzed via quantitative real-time PCR. Panels A and C show the relative fold-induction of mRNA for the gene Cytochrome 1a1, calculated using each sample's GAPDH expression, and then normalized to vehicle-treated controls via the delta-delta CT method; panels B and D are the same experiments, plotting Cytochrome 1b1. A one-way ANOVA test was used with Dunnet's correction for multiple comparisons to assess statistical significance between R-MT-treated and untreated cells. These data are the calculated average of four independent experiments using two independent MSC samples. E. MSCs were cultured in the presence of racemic 1MT (R-MT), or the pure enantiomer (L)-MT or (D)-MT at varying doses: (0.1 μM to 5000 μM). After 24h, cells were harvested, lysed and total RNA was extracted, converted to cDNA and analyzed via quantitative real-time PCR. **Panel E** shows the relative fold-induction of mRNA for the gene Cytochrome 1a1, calculated using each sample's GAPDH expression, and then normalized to vehicle-treated controls. Each treatment condition was fitted to a linear regression model, which were then compared by F-test to assess differences in line slope (P=0.009). **(F)** Panel F shows data from the same experiments as Panel E, plotting the relative fold-induction of mRNA for the gene Cytochrome 1b1, calculated using each sample's GAPDH expression, and then normalized to vehicle-treated controls. Each treatment condition was fitted to a linear regression model, which were then compared by F-test to assess differences in line slope (P=0.003). Panels E and F are summary data for nine experiments using two independent MSC samples.

We used a fixed time point to further explore the 1MT-mediated mRNA-induction of Cyp1a1 and Cyp1b1 [8, 20, 21] using clinically-relevant ranges of 1MT concentrations, with three different enantiomeric preparations. In current clinical trials with 1MT, patients are dosed orally up to 2000mg, achieving peak plasma concentrations of 1200 ng/ml (5.5 μM) [24, 25], and *in vitro* studies use 1mM dosing to inhibit IDO activity [2, 3]. As different publications explore different enantiomers of 1MT for IDO activity, we sought to assess if these three preparations would show different AHR activity profiles, at concentrations ranging from 5000 μM to 0.1 μM (Figure 3E–3F). Untreated controls were included in each experiment, and Fold-Induction of each cytochrome gene was calculated as above. These data were fitted to linear regression models, which were then compared for difference in slope, affording statistically-significant p-values, suggesting the racemic mixture (R-MT) may be more AHR-bioactive than either of the pure enantiomers. Taken together, these results indicate that over a variety of sub-clinical and clinical doses, all three mixtures of 1MT can induce the canonical AHR-driven response.

### Interferon-γ licensing of MSCs does not modify AHR response

**Figure 4:**
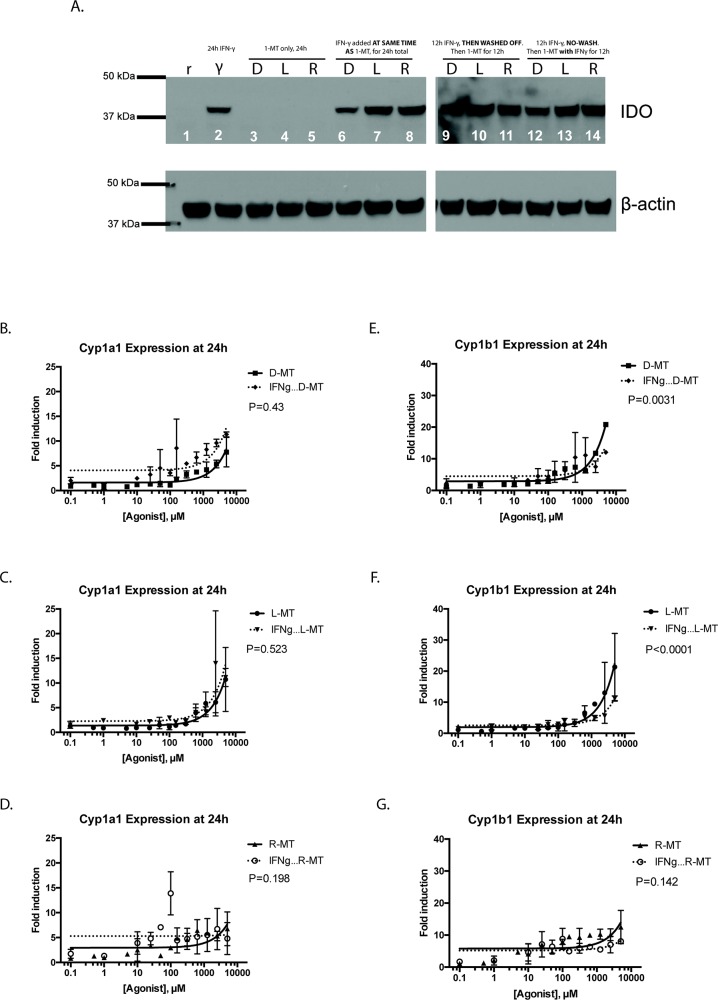
Interferon-γ licensing of MSCs and AHR response **(A)** Resting MSCs (r) and IFN-γ stimulated (γ) MSCs were analyzed for IDO expression after pre-stimulation with a fixed dose of IFN-γ (50 ng/ml), followed by treatment with a fixed 1mM dose of 1MT. Figure represents the immunoblotting results of a single membrane that was first blotted for IDO1, then stripped, re-blocked and probed for actin. Lanes 1-5 represent mono-treated cells. Lanes 6-8 represent 1MT and IFN-γ co-treatment; lanes 9-11 are an IFN-γ pre-stimulation, a PBS wash then 1MT alone. Lanes 12-14 represent a mono-treatment of IFN-γ, followed by 1MT co-treatment. These are results from an experiment with one MSC sample, which was replicated three times. **(B, C, D)** MSCs were cultured for 24h in the presence of a variable dose (0.1 μM to 5000μM) of racemic 1MT (R-MT), or the sole enantiomer (L)-MT or (D)-MT. In parallel experiments, MSCs were given 12h of pre-stimulation with IFN-γ, followed by 24h of 1MT treatment, using the same dose-titration curve. After the 1MT treatments, cells were harvested, lysed and total RNA was extracted, converted to cDNA and analyzed via quantitative real-time PCR. Panel B shows the relative fold-induction of mRNA for the gene Cytochrome 1a1, calculated using each sample's GAPDH expression, and then normalized to vehicle-treated controls. Each treatment condition was fitted to a linear regression model, which were then compared by F-test to assess for differences in line slope. **(E, F, G. Panels E)** F and G show data from the same experiments as B, C, D, but plot fold-induction of Cytochrome 1b1. As above, each treatment condition was fitted to a linear regression model, which were then compared by F-test to assess for differences in line slope. These six panels are the summary data for nine experiments using two independent MSC samples.

Our initial experiments showed that resting MSCs, negative for the IDO protein, were able to demonstrate robust upregulation of the downstream AHR signaling pathway in response to 1MT. However, it is conceivable that IDO+ cells might occupy equivalents of 1MT in the active site of the IDO protein, leaving none available to activate the AHR response [26]. To address this, we pre-treated MSCs with IFN-γ for 24h, which is sufficient to induce robust IDO protein expression [16]. Following, the IFN-γ was washed off and cells were treated with a fixed dose (1mM) of the 1MT enantiomeric preparations. Figure 4A is an immunoblot demonstrating that the amount of IDO protein expressed by MSCs does not alter when cells were also treated with 1MT. Figure 4B-4G shows the induction of the cytochrome genes when IFN-γ pre-stimulation was followed by 1MT, at a variety of dose titrations. A peak in Cyp1a1 induction occurred for at 100μM for D-MT and R-MT, but one was not observed for L-MT until 2.5 mM. We used linear regression and found that IFN-γ licensing of MSCs does not consistently alter the magnitude of cytochrome enzyme induction to a significant degree. This pattern is particularly important to note near 5.5 μM, which is the plasma concentration seen in humans dosed therapeutically with 1MT [24, 25]. These data indicate that 1MT can activate the AHR-driven response in MSCs in a comparable fashion, irrespective of IDO expression.

### RNA-seq shows 1MT and TCDD activate similar gene sets

**Figure 5:**
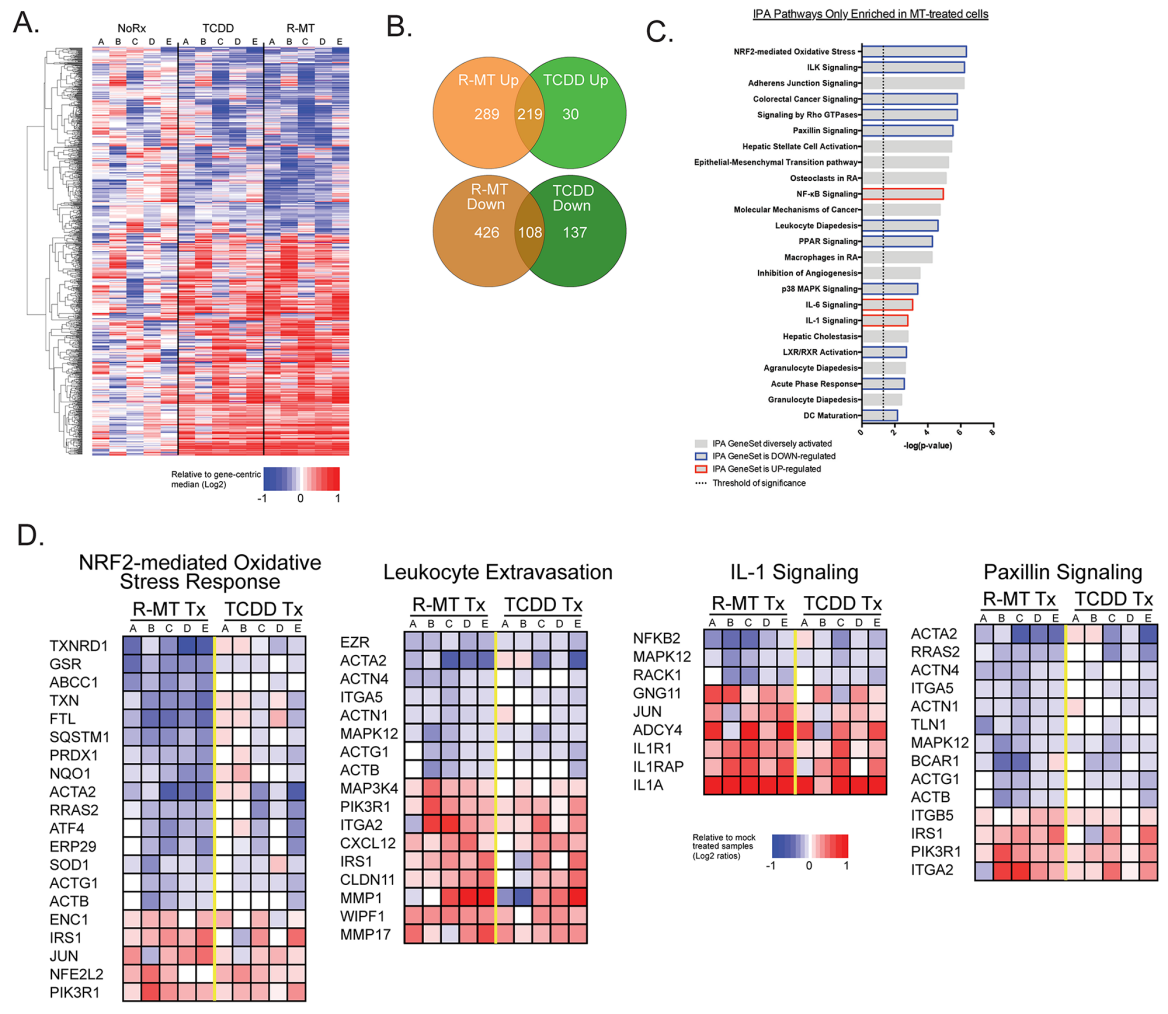
RNA-seq analysis of 1MT and TCDD treated MSCs **(A)** MSC samples (N=5) were cultured for 24h *in vitro* in the presence of R-MT (1 mM), TCDD (10 nM), or R10 vehicle (NoRx), and analyzed via mRNA-Seq. eat map displaying the union of all differentially-expressed genes (DEGs) found between control vehicle treated cells (NoRx) and TCDD treated cells or R-MT treated cells. DEGs were defined as +/−2-fold change and FDR <0.05. Hierarchical clustering was used to organize genes by expression pattern across samples. The color scale shown at bottom is defined as the ratio of each read-count to a gene-centric median, and maximum and minimums defined by a 2-fold upregulation (log2 = +1, red color) or downregulation (log2 = -1, blue color). **(B)** Venn diagrams showing degree of overlap of genes found to up-regulated or down-regulated in R-MT treated cells or TCDD treated cells relative to vehicle-treated controls. C. Ingenuity Pathway Analysis (IPA) was used to identify sets of functionally-related genes with statistically-significant enrichment in the genes differentially regulated by R-MT. **Panel C** is a curated list of 24 immunomodulatory pathways from the IPA databases found to be most significantly altered by R-MT, with the bar color indicating if net pathway activation was up, down, or more diversely activated, as determined by IPA Z-scores. **(D)** Figure 5D presents a heat map for four of the aforementioned 24 gene sets. To generate this heat map, each MSC sample was normalized to its own untreated control, allowing gene transcripts to be illustrated for up- or down-regulation on a per-MSC sample basis. They were subsequently scaled, whereby a +1.0 is a relative doubling from untreated samples and -1.0 is a relative halving. Patient samples were clustered separately along treatment parameters to compare the TCDD- and R-MT responses side-by-side.

Given that 1MT is known to be effective in cancer immunotherapy, we sought to use RNA profiling to identify novel immune signals induced by 1MT, and how those might be similar to the transcriptome of a verified AHR ligand. Five independent MSC samples were exposed for 24h to racemic 1MT, TCDD, or treated with vehicle only (NoRx); we then performed RNAseq analysis. We focused on differentially-expressed genes (DEGs) that were most significantly changed upon treatment with R-MT or TCDD. Hierarchical clustering was used to organize genes by expression pattern across samples. Figure 5A is a heat map representing the union of all DEGs found between the three conditions. Taken together, this heat map and its pattern suggests similar gene-activating signatures by R-MT and TCDD, especially when compared to sample-matched untreated controls. The Venn diagram in Figure 5B represents the degree of overlap for genes found to be up-regulated or down-regulated in R-MT-treated cells or TCDD-treated cells relative to controls. Among the up-regulated genes, we noted Cyp1a1 and Cyp1b1 (Supplementary Table 1) were both present, confirming an AHR-activating signature for both drugs; there were also 108 genes that were down-regulated in common (Figure 5B, Supplementary Table 1).

### IPA reveals a pro-inflammatory transcriptional signature for MSCs treated with 1MT

We next sought to identify the pathways that were uniquely affected by R-MT, but not by TCDD treatment (715 genes, Supplementarty Table 2. The 167 genes that were uniquely changed by TCDD are summarized in Supplementary Table 3). We performed an Ingenuity Pathway Analysis (IPA) on the genes from Supplementary Table 2, those uniquely affected by R-MT, and Figure 5C is a curated list of 24 immunomodulatory pathways most significantly altered, with the bar color indicating if net pathway activation was up, down, or more diversely activated. The most potently-activated pathway from this list was the Nrf2-mediated oxidative stress pathway, which was identified as being overall down-regulated as a result of R-MT treatment. Figure 5D presents a heat map for four of the aforementioned gene sets. To generate this heat map, each patient sample was normalized to its own untreated control, and IPA-pathway genes were assessed for up- or down-regulation on a per-patient basis. This heat map compares TCDD- and R-MT responses side-by-side. Key pathways are observed to be activated or down-regulated by both drugs, but in each case, R-MT was a more robust activator. These pathways are consistent with a cellular response poised towards pro-inflammatory infiltration of tumor tissues. Across all five samples, R-MT downregulates the Nrf2-mediated oxidative stress pathway, which is similar to the down-regulations observed in the paxillin pathway. Also of note was the net up-regulation of gene sets involved with the diapedesis of white blood cells, as well as the pro-inflammatory IL-1 pathway.

## DISCUSSION

Previous reports have indicated that the immunosuppressive effects of the IDO enzyme are due to the catabolism of tryptophan and the generation of secondary messenger metabolites. However, it remains unclear how those molecules may affect leukocytes, such as those that infiltrate a tumor. One such compound, kynurenine, was shown to have a net immunosuppressive effect on the proliferative capacity of inflammatory T cells [27], whereas others have been shown to activate the AHR and induce an pro-inflammatory response in cancer cells [23]. All of these tryptophan derivatives, including 1MT, contain an aromatic ring substituent. We hypothesized that the aromatic moieties in these compounds may rationalize their ability to serve as binding partner for the AHR, classically only appreciated as a receptor for aromatic hydrocarbon toxicants. We sought to characterize the effects of enantiomerically-pure and racemic mixtures because various human, murine and *in vitro* experiments have reported differential tumor clearance or IDO-inhibition for different enantiomeric preparations [24, 28–30]. On-going clinical trials use the enantiomerically-pure compound of D-MT [13, 24], which has been shown *in vitro* to be more effective at reversing tumor-mediated T cell suppression, and better *in vivo* synergy with conventional chemotherapy regimens [28]. Although many *in vitro* studies are conducted with a racemic mixture of R-MT [29], and it has been shown that the L enantiomer is a more effective inhibitor of IDO enzymatic activity [30]. Due to these conflicting reports, we tested the pure enantiomers as well as the racemic mixture, with some of our assays suggesting the racemic mixture was a stronger induce of the AHR response.

Although the present work has utilized a variety of indirect 1MT-to-AHR activation correlates, a direct ligand-binding assay will be necessary to validate the drug actually ligates the receptor. For example, there may exist an indirect middle actor(s) between the AHR response and treatment with 1MT. Ligand-binding studies such as the electromobility shift assay, as examined with free AHR protein and treatments with radiolabeled TCDD or 1MT would address this question [31].

Through a combination of biochemical, immunologic and bioinformatic methods, we demonstrate the efficacy of 1MT for cancer immunotherapy may be rationalized in part due to its AHR-activation. The tumor microenvironment contains malignant and non-malignant cells, as well as cells that may or may not express IDO. MSCs and their closely-related progeny can be mobilized to a growing tumor and participate in the formation of an immune suppressive microenvironment. Considering their innate ability to express IDO and constitutive expression of AHR, they provide a likely biological target for the pharmacological effects of 1MT. MSCs are touted as a therapeutic cell therapy tool, owing to their immune-suppressive or regenerative capabilities, but these same traits can become maladaptive in a tumor microenvironment. The process by which a tumor expands can be thought of as a chronic, non-healing wound [32]. The inflammatory milieu that attracts endogenous or local MSCs to repair damaged tissues can be usurped by a tumor, and the immune-suppressive effects of MSCs hijacked to help the tumor evade future attack by leukocytes. It is for these reasons that we sought to model the tumor microenvironment with the use of non-transformed MSCs, to understand the balance of inflammatory forces that can be targeted by adjuvant therapies like 1MT. Targeting IDO inhibition (or AHR activation) in a specifically-transformed cancer cell line simply would not afford the same immunotherapy-relevant insights that we have gained from using MSCs.

RNAseq profiling analyses revealed distinct pro-inflammatory signatures that were activated by 1MT, the most highly-significant of which was Nrf2-mediated oxidative stress. The Nrf2 pathway typically plays a protective role in tissues, mitigating inflammatory damage caused by environmental toxins. However, anti-inflammatory activity in a tumor microenvironment is not a positive-good phenomena; this anti-inflammatory signaling reflects the mechanisms of cancer immune-evasion [33], such as when tumor-infiltrating lymphocytes, or cell-based immunotherapeutics, are reprogrammed to ineffective regulatory cells [34]. The down-regulation of Nrf2 is interesting, as this gene is a known transducer of AHR-mediated signaling, not only for environmental toxins, but also for immune-modifying signals and hematopoietic cues [35]. Various reports have used chromatin-immunoprecipitation and sequencing to show that Nrf2 is an important regulator of anti-oxidant target genes, including HO-1, a key molecule that reduce cellular stresses from reactive oxygen species (ROS) [36–38]. Additionally, the 1MT-induced down regulation of Nrf2 helps explain how anti-inflammatory forces in a tumor microenvironment compete with infiltrating leukocytes to continually evade immune surveillance [34]. Similarly, overexpression of paxillin-family adhesion signaling proteins is a known signature of various tumor types [39, 40], so its down-regulation by 1MT is also consistent with a localized anti-tumor response. Overexpression of paxillin family members is a known signature of various tumor types [39, 40], so its net down-regulation by 1MT suggests this pathway may also be involved in the 1MT response. This is consistent with the role of 1MT in cancer immunotherapy, which by inhibition of IDO—or shown here as activating the AHR response—primes the immune system to fight tumors. Also of particular interest is the up-regulation of genes involved with extravasation by leukocytes, again consistent with an activated immune system, and tumor infiltration by lymphoid, myeloid or mesenchymal stromal cells.

More than half of the pathways enumerated in Figure 5 contain the pro-inflammatory cytokine IL-1a, and the ERK family kinase MAPK12 is also present at the same frequency. These genes were of interest as mechanisms by which tumor-associated cells could induce an inflammatory response, allowing infiltration by immune cells. Cross-comparisons with the Comparative Toxicogenomics Database revealed that MAPK12 is known to interact with benzopyrene, a toxicant in cigarette smoke, as well as DMBA, both of which are well-characterized AHR ligands known for potent toxicity in mammalian cells [41]. MAPK12 is also known for transducing signals related to cisplatin, etoposide and tamoxifen, three widely-used chemotherapeutic drugs [41]. To strengthen the association that MAPK12 may be transducing 1MT and AHR signals, we developed an in silico search algorithm to identify possible AHR response elements upstream of this putative AHR target gene. Our approach is modeled after a 2010 publication which utilized RNA-seq coupled with in silico bioinformatics to identify AHRE in target gene promoters, to putatively define them as downstream regulators [42]. Using this technique, Perdew et al. showed the 10kb-promoter region of the pro-inflammatory cytokine IL-6 contained an AHRE (GCGTG), rationalizing how a synthetic AHR ligand might stimulate the immune system. Notably, our own RNA-seq data reinforces these findings, as we noted an up-regulation of the IL-6 pathway in our R-MT transcriptome pathway analyses (Figure 5C). When we used this same scan-and-score algorithm to analyze the 10kb-promoter regions of MAPK12, we identified ten hypothetical AHR binding sites with sequence GCGTG. Similarly, the pro-inflammatory cytokine IL-1a contained two possible AHR binding sites with GCGTG.

The overlapping signals elicited by AHR toxicants (TCDD), as well as drugs in common usage but with incomplete understanding of their mechanisms of action (1MT) indicate possible steps forward in drug development. Importantly, we cannot be guided by traditional understanding of 1MT (as solely an IDO-inhibitor). It will be important to identify which types of AHR-activating ligands have pro-cancer effects (TCDD), which have anti-cancer effects (1MT), and what downstream activation panels will be most useful in screening compounds for bioactivity, via Cyp1a1/Cyp1b1 induction, Nrf2 repression or activation of MAPK12 or IL-1a.

The present work has utilized conventional biochemical and microscopy-based techniques to show that 1MT may act as an activator of the AHR pathway. However, beyond the identification of this signal, it has been important for us to define the downstream mechanisms by which 1MT may interact with AHR, in order to better characterize and pharmacologically exploit its cancer immunotherapy-augmenting abilities. By coupling RNAseq bioinformatics and in silico prediction modalities, we identified novel downstream actors that may rationalize how and why R-MT augments cancer immunotherapy. The finding that 1MT activates an AHR immune-activating signature—independent of IDO expression—suggests that this drug may have broader indications than previously anticipated. Taken together, this work lays the foundation for wider implementation of AHR-activating molecules, and the screening parameters that may guide further use of these molecules, to synergize immune-activation with conventional cancer treatment modalities.

## MATERIALS AND METHODS

### MSC isolation and culture

Human MSCs were isolated from bone marrow aspirates collected from the iliac crest of consenting volunteer subjects [43]. Bone marrow aspirates were diluted 1:2 with PBS and layered onto a Ficoll density gradient to isolate mononuclear cells. The cells were centrifuged at 400 × g for 20 min and thereafter plated in complete human MSC medium (α-MEM with L-glutamate, 10% human platelet lysate, 100 U/ml penicillin/streptomycin (Corning International, Corning, NY)) at 200,000 cells/cm^2^. Non-adherent hematopoietic cells were removed by changing the medium after 3 d of culture, and MSCs were allowed to expand for 7 d. Thereafter, the cells were passaged weekly and reseeded at 1000 cells/cm^2^. After the third passage, the MSC cultures were assayed by flow cytometric analysis for the absence of CD45^+^ and CD31^+^ contaminating cells and expression of CD44, CD73, CD90, and CD105 (BD Biosciences, San Jose, CA). Flow cytometry was performed using a FACSCanto II (BD Biosciences, San Jose, CA) and FlowJo software v9.6 (TreeStar, Ashland, OR). All assays were performed using MSCs between passages 3 and 6. Although culture-expanded in α-MEM, all subsequent tissue culture experimental work was performed in R10 (RPMI 1640 with L-glutamate plus 100 U/ml penicillin/streptomycin, and 10% fetal calf serum) (Corning International, Corning, NY). All cell culture work was performed in standard conditions in a tissue incubator at 37°C in 5% CO2 and 95% air.

### Immunoblotting

Approximately 1 million MSCs were harvested from a single 75-cm^2^ flask at 80% confluency. Cells had been treated for 12h with 50 ng/ml recombinant human IFN-γ (Invitrogen, Carlsbad, CA), and/or 1-methyl-DL-tryptophan, 1-methyl-D-tryptophan, or 1-methyl-L-tryptophan (Sigma-Aldrich, St. Louis, MO). Whole-cell protein lysates were run in a 4-20% polyacrylamide gel electrophoresis apparatus and then transferred to PVDF membrane, which was blocked in 5% non-fat milk in Tris-buffered saline + 0.05% Tween-20. Protein was detected using primary rabbit anti-human IDO1 (1:1000; EMD Millipore Corporation, Billerica, MA), primary mouse anti-human AHR (1:1000; ThermoFisher, Waltham, MA) or primary rabbit anti-human β-actin (1:1000; Cell Signaling Technology, Danvers, MA), and secondary horseradish peroxide-coupled goat anti-rabbit IgG h + l (1:10,000; Bethyl Laboratories, Montgomery, TX). ECL detection system (Amersham Pharmacia Biotech, Piscataway, NJ) was used to detect immunoreactive blots.

### q-RT-PCR analysis

MSCs cultured in the presence or absence of tryptophan derivatives or known AHR agonists were analyzed using quantitative Real-Time PCR. Total RNA was extracted and depleted of genomic DNA using the RNeasy plus mini kit (QIAGEN, Hilden, Germany). Normalized RNA was converted cDNA using Quantitect Reverse Transcription kit (QIAGEN, Hilden, Germany). Perfecta Sybr Green Fast Mix (Quanta Biosciences, Beverly, MA) real-time PCR was performed with the following primer pairs, listed with the forward primer followed by the reverse primer: GAPDH: 5′-CTC-TCT-GCT-CCT-CCT-GTT-CGA-C-3′; 5′-TGA-GCG-ATG-TGG-CTC-GGC-T-3′. Cyp1b1: 5′-GCT-GCA-GTG-GCT-GCT-CCT-3′; 5′-CCC-ACG-ACC-TGA-TCC-AAT-TCT-3′. Cyp1a1: 5′-CAC-CAT-CCC-CCA-CAG-CAC-3′; 5′-ACA-AAG-ACA-CAA-CGC-CCC-TT-3′. An ABI 7500 fast real-time PCR system thermal cycler (ThermoFisher, Waltham, MA) was used for amplification and the Δ-Δ C_T_ method was employed to calculate the fold change in expression [44]. Data are presented as normalized fold-induction above contemporaneously vehicle-treated controls.

### Immunofluorescence microscopy

In a twelve-welled tissue culture plate, 50,000 MSCs were plated onto glass coverslips and allowed to adhere overnight. Media was aspirated and replaced with R10 with/without indicated AHR testing ligand. Drugs: TCDD: 10nM (Supelco, St. Louis, MO), L-MT, D-MT, R-MT all at 1mM (Sigma Aldrich, St. Louis, MO). Cells were treated for 5h, after which media was aspirated and cells were fixed with 4% paraformaldehyde in PBS, then quenched with 50mM NH_4_Cl. Cells were permeabilized with 0.2% Triton and stained for AHR protein (1:100, ThermoFisher, Waltham, MA) diluted in 3% BSA in PBS (Sigma Aldrich, St. Louis, MO). Slips were kept overnight at 4°C in a humid chamber, washed with PBST, then stained (1:500) with a goat-derived anti-mouse secondary antibody with DyLight-488 (ThermoFisher, Waltham, MA). Isotype-control was a non-specific primary murine-derived IgG1 (BD Biosciences, San Jose, CA), followed by the same secondary. Glass slips were affixed to microscope slides using DAPI-containing VectaShield Dry-Curing mounting medium (Vector, Burlingame, CA) and then imaged using a confocal Zeiss SP8 microscope (Zeiss, Oberkochen, Germany). The Leica LASX software package (Leica, Wetzlar, Germany) was utilized by a treatment-blinded observer to delimit regions of interest, defined by the DAPI-stained nucleus. From these regions, the signal of Alexa488 was computed and normalized per square micron.

### Statistics

All graphical data for the project was analyzed using GraphPad Prism version 6.0 (GraphPad, La Jolla, CA), and the statistical tests of significance are noted where indicated, always using an alpha level set at 0.05.

### RNA-seq

RNA-Seq analyses were conducted at the Yerkes NHP Genomics Core on five independently-sourced MSC samples. Cells (1×10^5^) were plated into six-welled tissue culture plates in duplicates and treated with vehicle alone (R10), TCDD (10nM) or a racemic mixture of 1MT (1mM) for 24h. Total RNA was extracted from using QIAGEN RNEasy Mini kits (QIAGEN, Hilden, Germany) and RNA quality assessed using Agilent Bioanalyzer analysis. Polyadenylated transcripts were purified on oligo-dT magnetic beads, reverse transcribed using random hexamers, fragmented, and incorporated into barcoded complementary DNA libraries based on the Illumina TruSeq platform. Libraries were validated by microelectrophoresis, pooled, and sequenced on an Illumina HiSeq 1000 (101 bp) to an average read depth of 25 million [45]. 58,604 unique mRNA transcripts were identified in the data set. Reads were aligned to human RefSeq hg19 reference using STAR software (v2.3.0e) (http://code.google.com/p/rna-star) [46]. The RNAseq data discussed in this publication have been deposited in NCBI's Gene Expression Omnibus [47] and are accessible through GEO Series accession number GSE95072 (https://www.ncbi.nlm.nih.gov/geo/query/acc.cgi?acc=GSE95072).

### RNA-seq analyses

To examine differential gene expression in samples, estimates of gene-wise and isoform-wise expression levels for individual genes were performed using DESeq, which normalizes gene expression level estimates across samples and also corrects for nonuniformity in read distributions across each gene [48]. Each patient-sample-set included an untreated control, which was used to measure differential expression above baseline on a per-patient basis. Clustering by covariance PCA and visualization (i.e., heat maps) of expression data were performed in Partek Genomics Suite software (Partek Inc., St Louis, MO). Differentially expressed genes were analyzed for enriched gene families/pathways/protein interactions using Ingenuity Pathway Analysis (QIAGEN, Hilden, Germany). Gene set enrichment analysis was performed on the regularized log (rlog) expression table produced by DESeq2 employing a weighted enrichment statistic and Signal2Noise as the ranking metric and using 1000 phenotype permutations. The UCSC Genome browser, loaded with hg19, was used to analyzed the 10-kb promoter regions of MAPK12 and IL1a, prior to the first known exon, and a text-searching Python script employed to identify putative AHREs.

## SUPPLEMENTARY MATERIALS FIGURES AND TABLES









## References

[1] Lechner MG, Megiel C, Russell SM, Bingham B, Arger N, Woo T, Epstein AL (2011). Functional characterization of human CD33+ and CD11b+ myeloid-derived suppressor cell subsets induced from peripheral blood mononuclear cells co-cultured with a diverse set of human tumor cell lines. J Transl Med.

[2] Francois M, Romieu-Mourez R, Li M, Galipeau J (2012). Human MSC suppression correlates with cytokine induction of indoleamine 2,3-dioxygenase and bystander M2 macrophage differentiation. Mol Ther.

[3] Yu J, Du W, Yan F, Wang Y, Li H, Cao S, Yu W, Shen C, Liu J, Ren X (2013). Myeloid-derived suppressor cells suppress antitumor immune responses through IDO expression and correlate with lymph node metastasis in patients with breast cancer. J Immunol.

[4] Pallotta MT, Orabona C, Volpi C, Vacca C, Belladonna ML, Bianchi R, Servillo G, Brunacci C, Calvitti M, Bicciato S, Mazza EM, Boon L, Grassi F (2011). Indoleamine 2,3-dioxygenase is a signaling protein in long-term tolerance by dendritic cells. Nat Immunol.

[5] Mezrich JD, Fechner JH, Zhang X, Johnson BP, Burlingham WJ, Bradfield CA (2010). An interaction between kynurenine and the aryl hydrocarbon receptor can generate regulatory T cells. J Immunol.

[6] Eguchi H, Ikuta T, Tachibana T, Yoneda Y, Kawajiri K (1997). A nuclear localization signal of human aryl hydrocarbon receptor nuclear translocator/hypoxia-inducible factor 1 is a novel bipartite type recognized by the two components of nuclear pore-targeting complex. J Biol Chem.

[7] Smith KJ, Murray IA, Tanos R, Tellew J, Boitano AE, Bisson WH, Kolluri SK, Cooke MP, Perdew GH (2011). Identification of a high-affinity ligand that exhibits complete aryl hydrocarbon receptor antagonism. J Pharmacol Exp Ther.

[8] Chi AC, Appleton K, Henriod JB, Krayer JW, Marlow NM, Bandyopadhyay D, Sigmon RC, Kurtz DT (2009). Differential induction of CYP1A1 and CYP1B1 by benzo[a]pyrene in oral squamous cell carcinoma cell lines and by tobacco smoking in oral mucosa. Oral Oncol.

[9] Pinpin L, Han C, Tsai WT, Wu MH, Liao YS, Chen JT, Su JM (2003). Overexpression of aryl hydrocarbon receptor in human lung carcinomas. Toxicol Pathol.

[10] McFadyen MC, Rooney PH, Melvin WT, Murray GI (2003). Quantitative analysis of the Ah receptor/cytochrome P450 CYP1B1/CYP1A1 signalling pathway. Biochem Pharmacol.

[11] Hao N, Whitelaw ML (2013). The emerging roles of AhR in physiology and immunity. Biochem Pharmacol.

[12] Nguyen NT, Kimura A, Nakahama T, Chinen I, Masuda K, Nohara K, Fujii-Kuriyama Y, Kishimoto T (2010). Aryl hydrocarbon receptor negatively regulates dendritic cell immunogenicity via a kynurenine-dependent mechanism. Proc Natl Acad Sci U S A.

[13] NewLinkGeneticsCorp (2017). Study of the IDO Pathway Inhibitor, Indoximod, and Temozolomide for Pediatric Patients With Progressive Primary Malignant Brain Tumors. ClinicalTrials.gov.

[14] Lu Y, Giver C, Sharma A, Li JM, Darlak KA, Owens LM, Roback JD, Galipeau J, Waller EK (2012). “IFN-γ and indoleamine 2,3-dioxygenase signaling between donor dendritic cells and T cells regulates graft versus host and graft versus leukemia activity. Blood.

[15] Wuchter P, Bieback K, Schrezenmeier H, Bornhauser M, Muller LP, Bonig H, Wagner W, Meisel R, Pavel P, Tonn T, Lang P, Muller I, Renner M (2015). Standardization of Good Manufacturing Practice-compliant production of bone marrow-derived human mesenchymal stromal cells for immunotherapeutic applications. Cytotherapy.

[16] Romieu-Mourez R, Francois M, Boivin MN, Stagg J, Galipeau J (2007). Regulation of MHC class II expression and antigen processing in murine and human mesenchymal stromal cells by IFN-, TGF-, and cell density. J Immunol.

[17] Tkachenko A, Henkler F, Brinkmann J, Sowada J, Genkinger D, Kern C, Tralau T, Luch A (2016). The Q-rich/PST domain of the AHR regulates both ligand-induced nuclear transport and nucleocytoplasmic shuttling. Sci Rep.

[18] Davarinos N, Pollenz R (1999). Aryl Hydrocarbon receptor imported into the nucleus following ligand binding is rapidly degraded via the cytosplasmic proteasome following nuclear export. J Biol Chem.

[19] Tsuji N, Fukuda K, Nagata Y, Okada H, Haga A, Hatakeyama S, Yoshida S, Okamoto T, Hosaka M, Sekine K, Ohtaka K, Yamamoto S, Otaka M (2014). The activation mechanism of the aryl hydrocarbon receptor (AhR) by molecular chaperone HSP90. FEBS Open Bio.

[20] Cui Z, Li P, Liu J, Liu S (2008). [Induction of CYP1A1 expression of H4IIE cell after treated with the water organic pollutants from the Yangtze River and Jialing River]. [Article in Chinese]. Wei Sheng Yan Jiu.

[21] Elshenawy OH, El-Kadi AO (2015). Modulation of aryl hydrocarbon receptor-regulated enzymes by trimethylarsine oxide in C57BL/6 mice: in vivo and in vitro studies. Toxicol Lett.

[22] Wincent E, Kubota A, Timme-Laragy A, Jonsson ME, Hahn ME, Stegeman JJ (2016). Biological effects of 6-formylindolo[3,2-b]carbazole (FICZ) in vivo are enhanced by loss of CYP1A function in an Ahr2-dependent manner. Biochem Pharmacol.

[23] DiNatale BC, Murray IA, Schroeder JC, Flaveny CA, Lahoti TS, Laurenzana EM, Omiecinski CJ, Perdew GH (2010). Kynurenic acid is a potent endogenous aryl hydrocarbon receptor ligand that synergistically induces interleukin-6 in the presence of inflammatory signaling. Toxicol Sci.

[24] Soliman HH, Minton SE, Han HS, Ismail-Khan R, Neuger A, Khambati F, Noyes D, Lush R, Chiappori AA, Roberts JD, Link C, Vahanian NN, Mautino M (2016). A phase I study of indoximod in patients with advanced malignancies. Oncotarget.

[25] Dunham RM, Gordon SN, Vaccari M, Piatak M, Huang Y, Deeks SG, Lifson J, Franchini G, McCune JM (2013). Preclinical evaluation of HIV eradication strategies in the simian immunodeficiency virus-infected rhesus macaque: a pilot study testing inhibition of indoleamine 2,3-dioxygenase. AIDS Res Hum Retroviruses.

[26] Huang Q, Zheng M, Yang S, Kuang C, Yu C, Yang Q (2011). Structure-activity relationship and enzyme kinetic studies on 4-aryl-1H-1,2,3-triazoles as indoleamine 2,3-dioxygenase (IDO) inhibitors. Eur J Med Chem.

[27] Chinnadurai R, Copland IB, Patel SR, Galipeau J (2014). IDO-independent suppression of T cell effector function by IFN-gamma-licensed human mesenchymal stromal cells. J Immunol.

[28] Hou DY, Muller AJ, Sharma MD, DuHadaway J, Banerjee T, Johnson M, Mellor AL, Prendergast GC, Munn DH (2007). Inhibition of indoleamine 2,3-dioxygenase in dendritic cells by stereoisomers of 1-methyl-tryptophan correlates with antitumor responses. Cancer Res.

[29] Jia L, Schweikart K, Tomaszewski J, Page JG, Noker PE, Buhrow SA, Reid JM, Ames MM, Munn DH (2008). Toxicology and pharmacokinetics of 1-methyl-d-tryptophan: absence of toxicity due to saturating absorption. Food Chem Toxicol.

[30] Qian F, Villella J, Wallace PK, Mhawech-Fauceglia P, Tario JD, Andrews C, Matsuzaki J, Valmori D, Ayyoub M, Frederick PJ, Beck A, Liao J, Cheney R (2009). Efficacy of levo-1-methyl tryptophan and dextro-1-methyl tryptophan in reversing indoleamine-2,3-dioxygenase-mediated arrest of T-cell proliferation in human epithelial ovarian cancer. Cancer Res.

[31] Soshilov AA, Denison MS (2014). DNA Binding (Gel Retardation Assay) Analysis for Identification of Aryl Hydrocarbon (Ah) Receptor Agonists and Antagonists. In: Caldwell GW YZ, ed. Optimization in Drug Discovery: In Vitro Methods.

[32] Shi Y, Du L, Lin L, Wang Y (2017). Tumour-associated mesenchymal stem/stromal cells: emerging therapeutic targets. Nat Rev Drug Discov.

[33] Swann JB, Smyth MJ (2007). Immune surveillance of tumors. J Clin Invest.

[34] Beavis PA, Slaney CY, Kershaw MH, Gyorki D, Neeson PJ, Darcy PK (2016). Reprogramming the tumor microenvironment to enhance adoptive cellular therapy. Semin Immunoly.

[35] Ahmed SM, Luo L, Namani A, Wang XJ, Tang X (2016). Nrf2 signaling pathway: pivotal roles in inflammation. Biochim Biophys Acta.

[36] Zhu J, Wang H, Chen F, Fu J, Xu Y, Hou Y, Kou HH, Zhai C, Nelson MB, Zhang Q, Andersen ME, Pi J (2016). An overview of chemical inhibitors of the Nrf2-ARE signaling pathway and their potential applications in cancer therapy. Free Radic Biol Med.

[37] Li L, Dong H, Song E, Xu X, Liu L, Song Y (2014). Nrf2/ARE pathway activation, HO-1 and NQO1 induction by polychlorinated biphenyl quinone is associated with reactive oxygen species and PI3K/AKT signaling. Chem Biol Interact.

[38] Li N, Alam J, Venkatesan MI, Eiguren-Fernandez A, Schmitz D, Di Stefano E, Slaughter N, Killeen E, Wang X, Huang A, Wang M, Miguel AH, Cho A (2004). Nrf2 is a key transcription factor that regulates antioxidant defense in macrophages and epithelial cells: protecting against the proinflammatory and oxidizing effects of diesel exhaust chemicals. J Immunol.

[39] Kanteti R, Batra SK, Lennon FE, Salgia R (2016). FAK and paxillin, two potential targets in pancreatic cancer. Oncotarget.

[40] Mowers EE, Sharifi MN, Macleod KF (2016). Novel insights into how autophagy regulates tumor cell motility. Autophagy.

[41] Davis AP, Grondin CJ, Johnson RJ, Sciaky D, King BL, McMorran R, Wiegers J, Wiegers TC, Mattingly CJ (2017). The comparative toxicogenomics database: update 2017. Nucleic Acids Res.

[42] DiNatale BC, Schroeder JC, Francey LJ, Kusnadi A, Perdew GH (2010). Mechanistic insights into the events that lead to synergistic induction of interleukin 6 transcription upon activation of the aryl hydrocarbon receptor and inflammatory signaling. J Biol Chem.

[43] Chinnadurai R, Copland IB, Garcia MA, Petersen CT, Lewis CN, Waller EK, Kirk AD, Galipeau J (2016). Cryopreserved mesenchymal stromal cells are susceptible to T-cell mediated apoptosis which is partly rescued by IFNgamma licensing. Stem Cells.

[44] Livak KJ, Schmittgen TD (2001). Analysis of relative gene expression data using real-time quantitative PCR and the 2(-delta delta C(T)) method. Methods.

[45] Marioni JC, Mason CE, Mane SM, Stephens M, Gilad Y (2008). RNA-seq: an assessment of technical reproducibility and comparison with gene expression arrays. Genome Res.

[46] Dobin A, Davis CA, Schlesinger F, Drenkow J, Zaleski C, Jha S, Batut P, Chaisson M, Gingeras TR (2013). STAR: ultrafast universal RNA-seq aligner. Bioinformatics.

[47] Edgar R, Domrachev M, Lash AE (2002). Gene Expression Omnibus: NCBI gene expression and hybridization array data repository. Nucleic Acids Res.

[48] Golden MR, Ashley-Morrow R, Swenson P, Hogrefe WR, Handsfield HH, Wald A (2005). Herpes simplex virus type 2 (HSV-2) western blot confirmatory testing among men testing positive for HSV-2 using the focus enzyme-linked immunosorbent assay in a sexually transmitted disease clinic. Sex Transm Dis.

