# Supplementary material for: The IDO inhibitor 1-methyl tryptophan activates the aryl hydrocarbon receptor response in mesenchymal stromal cells
Source: Oncotarget. 2017 Aug 10;8(54):91914–27. doi: 10.18632/oncotarget.20166 (PMC5696151; doi:10.18632/oncotarget.20166)
Supplement: Supplementary file 1 [file oncotarget-08-91914-s001.pdf]

The IDO inhibitor 1-methyl tryptophan activates the aryl hydrocarbon receptor response in mesenchymal stromal cells

SUPPLEMENTARY MATERIALS

Supplementary Table 1: A listing of the genes that were identified in the RNAseq analysis for Figure 5 to be differentially expressed upon treatment with R-MT and also by TCDD

See Supplementary File 1

Supplementary Table 2: A listing of the genes that were identified in the RNAseq analysis for Figure 5 to be differentially expressed ONLY upon treatment with R-MT

See Supplementary File 2

Supplementary Table 3: A listing of the genes that were identified in the RNAseq analysis for Figure 5 to be differentially expressed ONLY upon treatment with TCDD

See Supplementary File 3
